# Molecular mechanisms for the inheritance of acquired characteristics—exosomes, microRNA shuttling, fear and stress: Lamarck resurrected?

**DOI:** 10.3389/fgene.2014.00133

**Published:** 2014-05-15

**Authors:** John Smythies, Lawrence Edelstein, Vilayanur Ramachandran

**Affiliations:** ^1^Department of Psychology, Center for Brain and Cognition, University of CaliforniaSan Diego, La Jolla, CA, USA; ^2^Medimark CorporationDel Mar, CA, USA

**Keywords:** transgenerational inheritance, epigenetics, microRNAs, exosomes, epididymis, sperm, Lamarck

## Introduction

Jean-Baptiste Lamarck (1744–1829) was the author of the first scientific hypothesis about the inheritance of characteristics. He proposed that the changing environment causes changes in bodily organs (e.g., the giraffe's elongating neck in response to the growth of taller trees) that are transmitted to the offspring. Darwin's theory and the discovery of DNA led to the eclipse of this theory. Recently, however, his ideas in a modified form have received a degree of support. It is now recognized that environmental epigenetic factors, that leave the sequence of DNA base pairs unaltered, nevertheless affect the relation between the genotype and the phenotype by modulating the expression of genes at various levels that include methylation and acetylation of DNA and histones, as well as microRNAs that modulate mRNA activity.

This paper will explore two possible molecular mechanisms, based on microRNAs and exosomes, which may contribute to the inheritance of acquired characteristics. It will not be concerned directly with other mechanisms proposed in this field, such as the epigenetic reprogramming of the developing germ line, including epimutations, which may be responsible for some instances of inheritance. Stringer et al. (2013) have recently reviewed the Byzantine complexity of this field (also see Dias and Ressler, 2014). We propose to explore two additional (non-competitive) fields presented in the form of two hypotheses. The first involves short range exosome signaling and the second involves long range exosome signaling.

## The facts

Sperm carry not only DNA to the ovum but a wide variety of RNAs. These include mRNAs, microRNAs and piRNAs (Kumar et al., 2013). The authors suggest that these RNAs may play a role in embryo development by regulating the expression of various genes. These microRNAs reach the developing sperm in the epididymis, where they are transported by exosomes from the epididymal epithelium (Sullivan et al., 2005). MicroRNA repertoires contained within epididymal exosomes differ from those of their parent epithelial cells (Belleannée et al., 2013). Hossain et al. (2012) have reviewed the contribution of microRNAs to germ cell differentiation, post-meiotic male germ cell function and growth, and development and maturation of oocytes through pertaining tightly-regulated gene expression. (Kawano et al., 2012) report the discovery of two novel microRNAs (spR-12 and spR-14) in sperm and ovulated unfertilized oocytes, present in one-cell embryos and maintained in preimplantation stages, but not at later differentiation stages. The authors go on to suggest that these findings offer a new perspective regarding a possibly important role for gamete-specific small RNAs in early embryogenesis. mRNA regulation is essential in germ cells and early embryos (Barckmann and Simonelig, 2013). Jodar et al. (2013) list the various types of non-coding RNAs in sperm and suggest that some of these may play a role in transgenerational inheritance.

In experiments using parental male mice stressed for 6 weeks before breeding, examination of the offspring both throughout puberty and in adulthood revealed a significant increase in the expression of nine specific sperm microRNAs resulting from the stress: miR-193-5p, miR-204, miR-29c, miR-30a, miR-30c, miR-32, miR-375, miR-532–3p, and miR-698 (Rodgers et al., 2013). An assessment of the top-predicted mRNA targets of the nine significantly increased miRNAs revealed those for DNMT3a (DNA methyltransferase 3a), a critical regulator of *de novo* DNA methylation important for imprinted genes, and two proteins involved in microRNA processing. The authors went on state that these genes, targeted by four of the nine up-regulated microRNAs, suggest that paternal stress may affect the offspring's hypothalamic-pituitary adrenal (HPA) axis by intervening in the epigenetic regulation of embryonic development. Furthermore, there is a low level of these microRNAs in unstressed animals. This entails that the scope for modulation is mainly to increase these abnormally low levels. Twenty-eight microRNAs were found to be differentially expressed in the sperm from male smokers vs. non-smokers Marczylo et al. (2012). Likewise, the sperm of insulin-resistant and normal mice show abnormal expression of 11 microRNAs (Fullston et al., 2013). These last two reported findings were shown to be expressed in subsequent generations.

## Hypothesis 1

### Short range exosome signaling

We suggest that parental stress up-regulates the production of the microRNAs specified above in the epididymis, via activation of the HPA axis. Other chemical signals, generated by such factors as tobacco usage and insulin resistance, may also modulate microRNA expression. Subsequently, these microRNAs are shuttled by exosomes to the sperm and conveyed to the ovum. Here, they may epigenetically up-regulate the sensitivity of the developing HPA system in the developing embryo during the formation of the HPA axis. The fact that the top-predicted target of the microRNAs detected by Rodgers et al. (2013) was a DNA methyltransferase may be significant in this context. Consequently, when the adult offspring is stressed, it would exhibit behaviors associated with having an HPA system with a lowered threshold for activation. This reaction would in turn lower the threshold for expression of particular stress-evoked miRNAs in its own epididymis and sperm, owing to a repeat of the mechanism described above. In turn, this would result in transmission of the characteristic to subsequent generations. The proposed lowering of the HPA axis threshold for expression could be a loss of canalization effect similar to loss of canalization effects in Drosophila (Gursky et al., 2011; Marques-Pita and Rocha, 2013). Such mechanisms of a repeated nature could serve to reinforce the changes seen through the generations rather than just repeat them.

Stress in early life induces the high expression of five miRNAs in the sperm of mice, one of which (miRNA-375) has been linked to stress and the regulation of metabolism. The F1 male's offspring (the F2 generation) showed depressive behaviors, as well as abnormal sugar metabolism and raised levels of the five miRNAs in their blood and hippocampus (Gapp et al., 2014). Interestingly the initial stressful experience did not affect the sperm miRNA in the F2 and F3 generations. This suggests that abnormal levels of the five miRNAs mediate the epigenetic transmission from the F1 to the F2 generations, whereas the epigenetic transmission from the F2 to subsequent generations may be mediated by downstream modifications of DNA methylation engineered by these miRNAs.

This hypothesis may be tested in the following manner. Input manipulations, which result in effects harmful to the organism, should modulate the pattern of sperm-transported microRNAs in a particular way [such as carrying a microRNA that targets the mRNA related to a DNA methyltransferase, as reported by Rodgers et al. (2013)]. In contrast, an input manipulation that results in an effect beneficial to the organism might be expected to modulate the pattern of sperm-transported microRNAs in a different way (such as the inclusion of a microRNA that targets a DNA demethylase; see also Dias and Ressler, 2014). Other experiments along similar lines seem possible.

## Hypothesis 2

### Long range exosome signaling

(Sahoo et al., 2014) present remarkable data, which suggest a role for exosomes released from a damaged heart as potential long-range intercellular communicators. These exosomes carry epigenetic-signaling molecules via the blood, which then activate stem cells in the bone marrow. Furthermore, other exosomes, which are released back into the blood from these reprogrammed stem cells, return to the heart and effectively reprogram its ischemic tissues, inducing protection and regeneration.

If we look further into the details of this mechanism some interesting points arise. The exosomes released from the ischemic heart muscle must bear on their external plasma membranes the cell recognition molecule (CRM–a lipoproteinpolysaccharide) that will recognize the complementary CRM on the surface of the stem cell to facilitate the binding and uptake of the exosome into the stem cell. Secondly, the stem cell's CRM must recognize only CRMs from injured heart muscle, or else it would be inundated by false hits from exosomes from normal cells. Thirdly, it would seem unlikely that cardiac muscle is the only bodily tissue, which utilizes this mechanism.

Could a similar system be in place with respect to the communication between the brain and cells in the male and female germ line? This would require cells in the distressed brain to secrete exosomes into the blood loaded with a diverse cargo of epigenetic material (Edelstein and Smythies, 2014; Smythies and Edelstein, 2013a,b). Exosomes freely cross the blood-brain barrier in both directions (Kalani et al., 2014; Pusic et al., 2014; Sampey et al., 2014). They would also require particular surface CRMs that would be recognized by CRMs on the surface of the germ cells. A mechanism for providing this may be as follows. The formation of exosomes by a cell involves specialization of the membrane of the multivesicular body that constructs exosomes into tetraspannin-enriched membrane domains (TEMs) (Gupta and Pulliam, 2014). One function of a TEM is to recruit specific proteins and other ligands on the TC membrane needed to enable CRM-based endocytosis by the specific target cell. Thus certain exosomes emitted by the donor cell could be targeted to the cells of the genome in this manner. This hypothesis could be tested by seeing if such CRM designated exosomes can be detected.

Going forward, we suggest the design of experiments to test these hypotheses, as, regardless of the outcome, the return will be worth the investment. Carolus Linnaeus might well agree in the context of his seminal study of Peloria (Gustaffson, 1979).

### Conflict of interest statement

The authors declare that the research was conducted in the absence of any commercial or financial relationships that could be construed as a potential conflict of interest.
